# Crossed Aphasia as a Manifestation of Glioblastoma

**DOI:** 10.7759/cureus.2239

**Published:** 2018-02-27

**Authors:** Samer G Zammar, Charles S Specht, Brad E Zacharia

**Affiliations:** 1 Department of Neurosurgery, Penn State Milton S Hershey Medical Center; 2 Pathology, Penn State Milton S Hershey Medical Center

**Keywords:** crossed aphasia, glioblastoma

## Abstract

Language and speech function is commonly accepted to be a heavily lateralized function. Greater than 95% of right-handed individuals have left hemispheric dominance for language, and reports in the literature of crossed aphasia (language deficits in a right-handed individual from right-sided pathology) are scant. We report the case of a 52-year-old woman presenting with crossed aphasia from a right temporal glioblastoma. We then expand on a discussion of crossed aphasia in the setting of brain tumors.

## Introduction

Aphasia is the failure to understand or produce language. Patients tend to show signs of difficulty speaking or understanding language. Reading, writing, as well as the use of manual sign language, may also be affected. Bramwell introduced the concept of crossed aphasia in 1899 [1] and defined it as the presence of aphasia after the involvement of the cerebrum ipsilateral to the dominant hand. As left-handed individuals tend to be less lateralized than right-handed people as regards language function, the concept of crossed aphasia is primarily utilized in right hemispheric lesions resulting in aphasia in right-handed patients [2]. This phenomenon is most often described in post-stroke patients and only rarely reported with neoplasms and other pathologies. We present the case of a 52-year-old woman presenting with crossed aphasia from a right temporal glioblastoma. We then expand the discussion of crossed aphasia in the setting of brain tumors.

## Case presentation

Presentation and imaging

**Figure 1:**
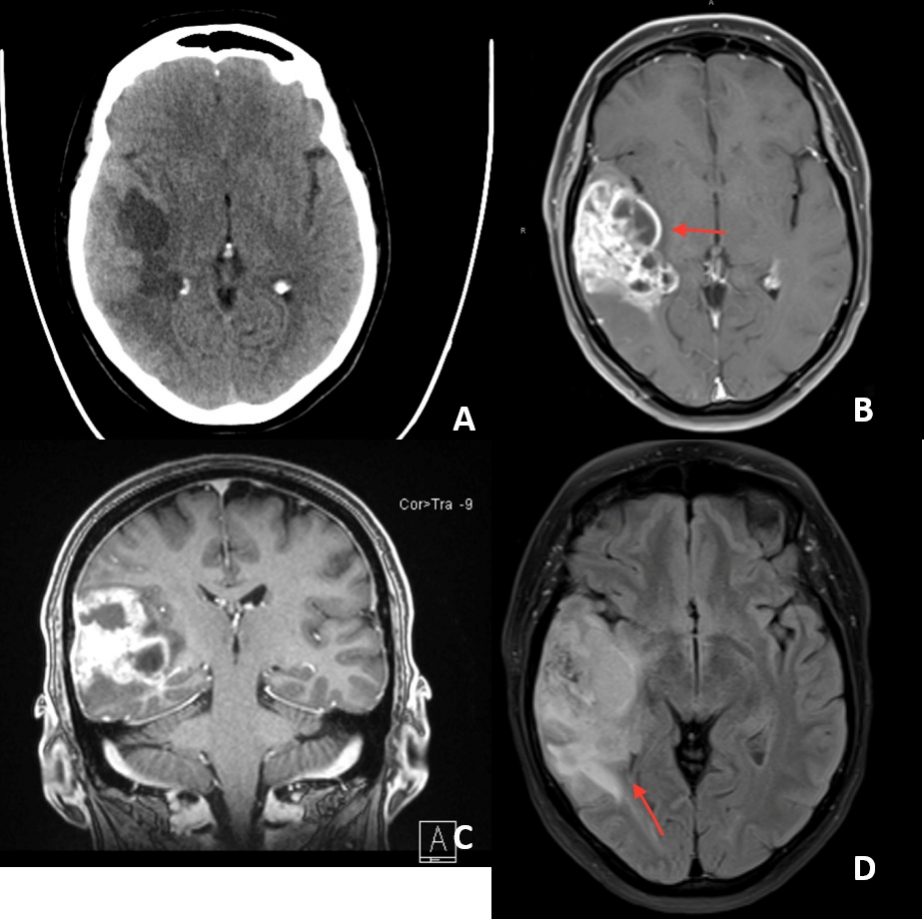
Imaging at emergency room admission A) Axial computed tomography (CT) scan showing hypodensity in the right hemisphere suggesting right middle cerebral artery stroke; B) Axial T1 magnetic resonance imaging (MRI) sequence with contrast showing mixed cystic and solid (arrow) enhancing right-sided temporal lesion concerning for brain neoplasm. The lesion involves the superior temporal gyrus anteriorly and all three temporal gyri posteriorly with minimal infiltration into the inferior most gray matter at the opercular border; C) Coronal section of the same sequence; D) Fluid-attenuated inversion recovery (FLAIR) sequence and the associated edema (arrow).

Our patient is a 52-year-old right-handed woman who initially presented to the emergency department with left face and left upper extremity numbness. On physical examination, the patient demonstrated a moderate expressive aphasia without evidence of a receptive component. The patient scored 100 on the Edinburgh Handedness Inventory indicating as strong right-handedness as possible. Computed tomography (CT) scan of the brain completed in the emergency department demonstrated a right-sided hypodensity concerning for a middle cerebral artery (MCA) stroke (Figure 1). Subsequent magnetic resonance imaging (MRI) of the brain with and without gadolinium contrast revealed an enhancing, mixed cystic and solid lesion in the right temporal lobe. The lesion’s characteristics were concerning for a primary glial neoplasm involving the superior temporal gyrus anteriorly and all three temporal gyri posteriorly with minimal infiltration into the inferior gray matter at the opercular border of the right postcentral gyrus (Figure 1).

**Figure 2:**
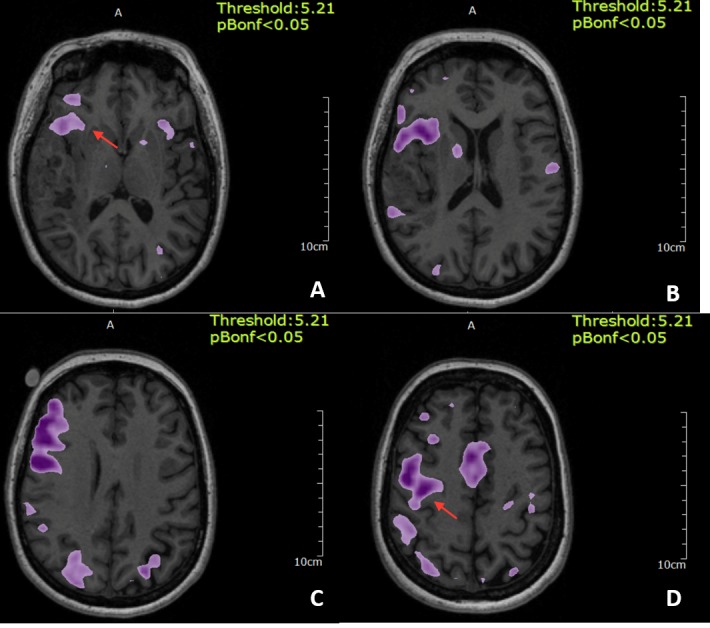
Functional magnetic resonance imaging (fMRI) for word generation A) Functional MRI for word generation which shows a cluster of activity seen in the right inferior frontal gyrus (arrow) separated from the tumor by the Sylvian fissure; B, C) Functional MRI also shows two separate clusters of activity in the right parietal lobe and the superior temporal gyrus which is very close to the tumor margin; D) Shows the activity extending to the adjacent right precentral gyrus.

A functional MRI for word generation revealed a large cluster of activity in the right inferior frontal gyrus, separated from the tumor by the Sylvian fissure, and extending into the inferior portion of the adjacent right precentral gyrus. There were two additional distinct clusters of activity in the right inferior parietal lobe and in the superior temporal gyrus, which were in close proximity to the tumor margin (Figure 2).

**Figure 3:**
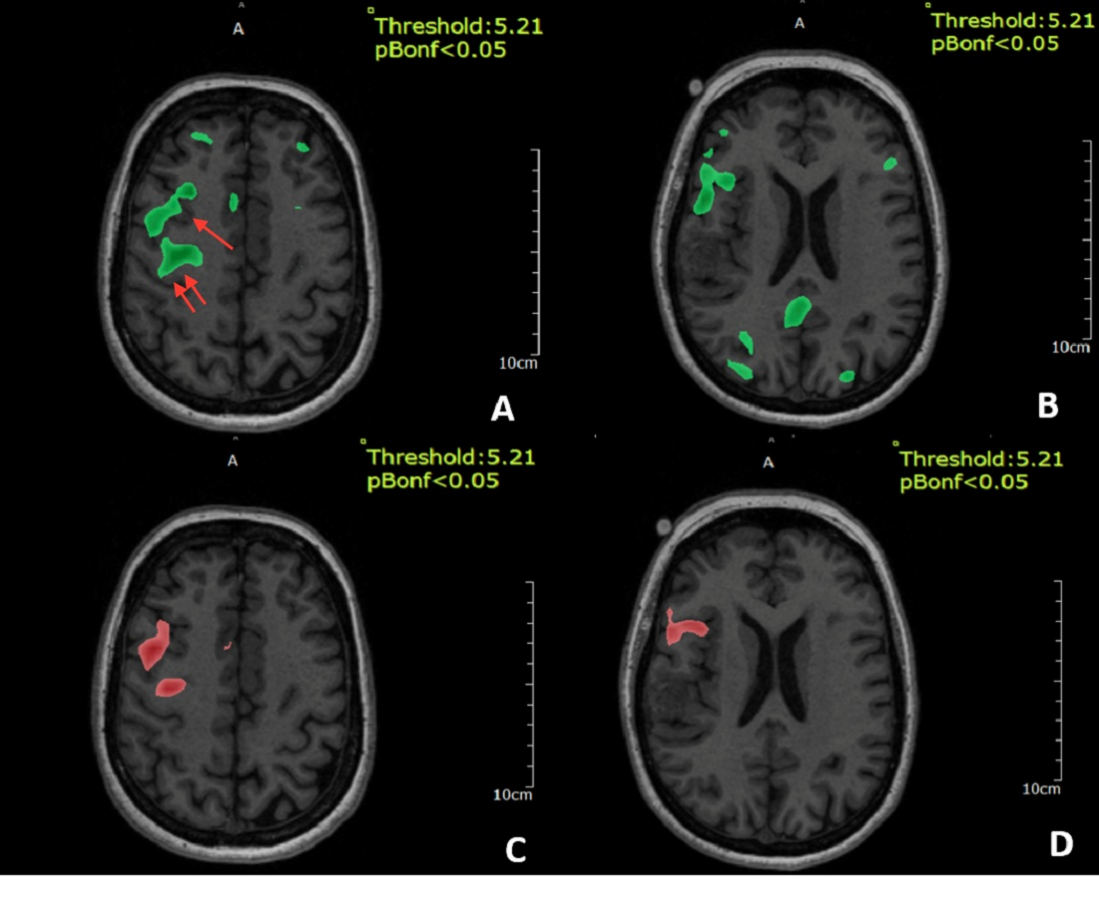
Functional MRI (fMRI) for verb generation A and B) show the functional MRI for sentence completion which reveal dominant activity in the right inferior frontal gyrus (arrow) and right precentral gyrus (double arrows), C) shows the functional MRI for verb generation, D) shows the functional MRI for word listening which is mainly confined to the left superior temporal gyrus.

**Figure 4:**
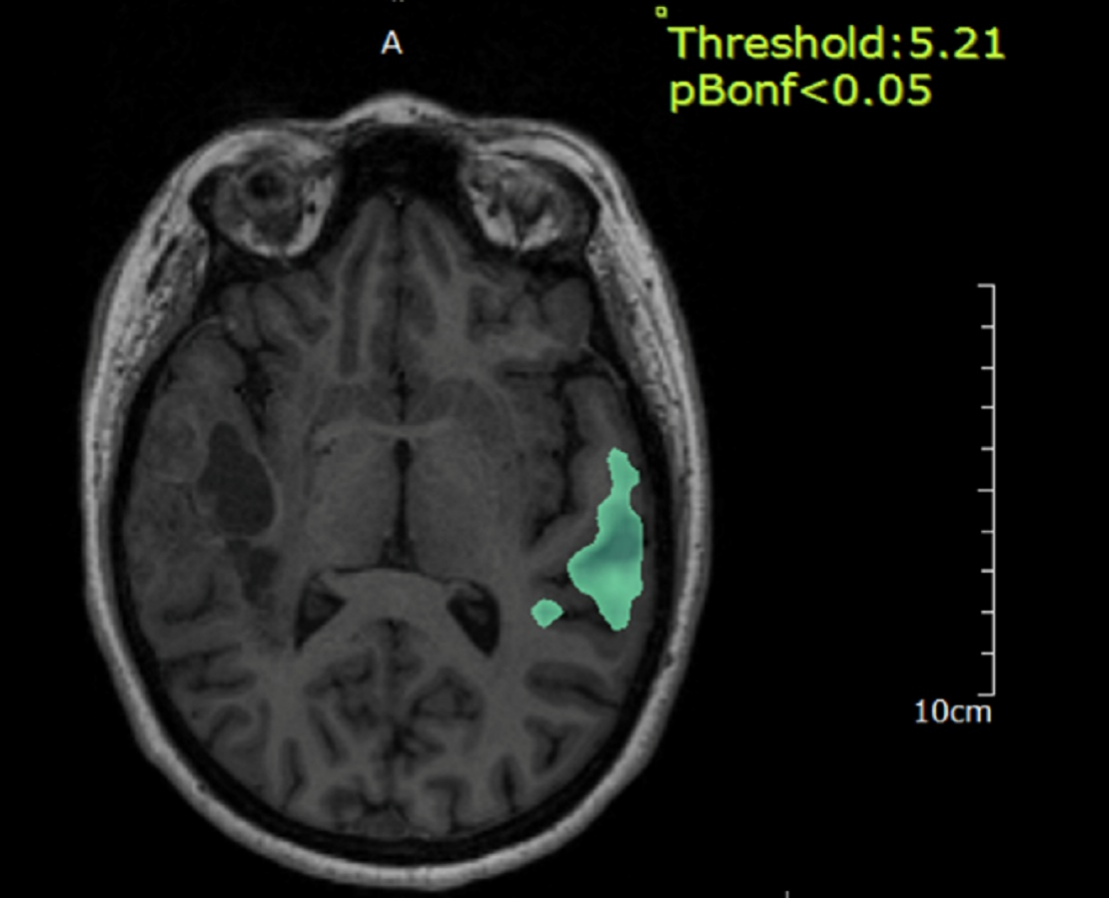
Functional magnetic resonance imaging (fMRI) for word listening Functional MRI for word listening, which is mainly confined to the left superior temporal gyrus.

Sentence completion and verb generation (Figure 3) tasks also showed dominant activity in the right inferior frontal gyrus and right precentral gyrus. Word listening was primarily confined to the left superior temporal gyrus (Figure 4).

Neuropsychological testing

Detailed neuropsychological testing was done preoperatively. The patient’s language was tested with the Controlled Oral Word Association test (COWA) and the Boston Naming Test (BNT). COWA includes two components that require patients to list all the words that they think of that begin with a certain letter and, in the second component, to list all the words that they can think of that belong in a certain category. On both the letter fluency task and the category fluency task, the patient’s performance was in the borderline range. The BNT requires the patients to name the common name of everyday objects. The patient was found to be within the mildly impaired range in this test. On other aspects of neuropsychological testing, the patient’s intelligence fell within the normal range, her memory domain ranged from average to profoundly impaired, and her attention and executive functioning were in the average to low ranges. Furthermore, the patient completed a self-report questionnaire indicating minimal anxiety and mild depression.

Surgery

To obtain a tissue diagnosis and for maximal safe tumor debulking, we proceeded with surgical resection. Given the results of her functional imaging and neuropsychological testing, we planned an awake right frontotemporal craniotomy with intraoperative neurophysiologic monitoring to maximize the safety and efficacy of our resection. The frontotemporal region was exposed and the tumor was identified with a combination of ultrasound and neuronavigation. After that, the patient was awakened, and we proceeded with functional mapping of the exposed frontotemporal region. Bipolar stimulation at 0.4 mA elicited speech arrest in the right frontal operculum; stimulation of the superior temporal gyrus resulted in difficulty with sentence completion. A region devoid of positive stimulation was identified and utilized as a foray into the tumor. The initial specimen was taken for frozen section pathology and was  consistent with a high-grade glial neoplasm. Interestingly, as tumor debulking proceeded, her language function appeared to improve from the preoperative baseline. The tumor appeared to have invaded the pia of the Sylvian fissure. We decided to leave a small rind of tumor in this region as there was a concern for potential damage to the Sylvian vasculature. The remainder of the resection proceeded to the functional boundaries, with the patient continuing routine language tasks.

Postoperative course

**Figure 5:**
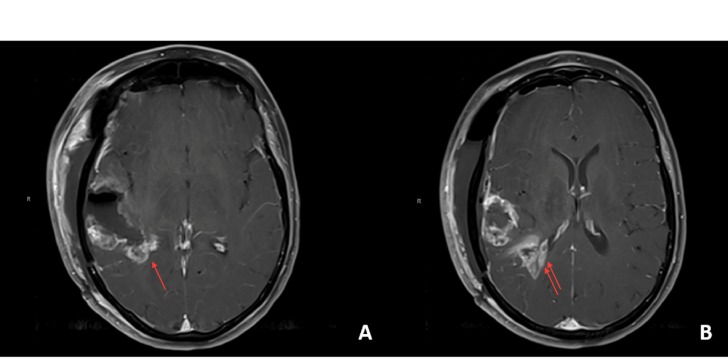
Postoperative magnetic resonance imaging (MRI) A) shows axial T1 MRI with contrast showing expected residual tumor along the Sylvian fissure (arrow) and B) in the posteromedial white matter adjacent to the atrium (double arrows).

Postoperatively, the patient was at her neurologic baseline with moderate word finding difficulty. She subsequently underwent adjuvant chemoradiation with temozolomide. Postoperative MRI demonstrated a radical subtotal resection, with expected persistent enhancement along the Sylvian fissure and adjacent to the atrium of the right ventricle (Figure 5).

**Figure 6:**
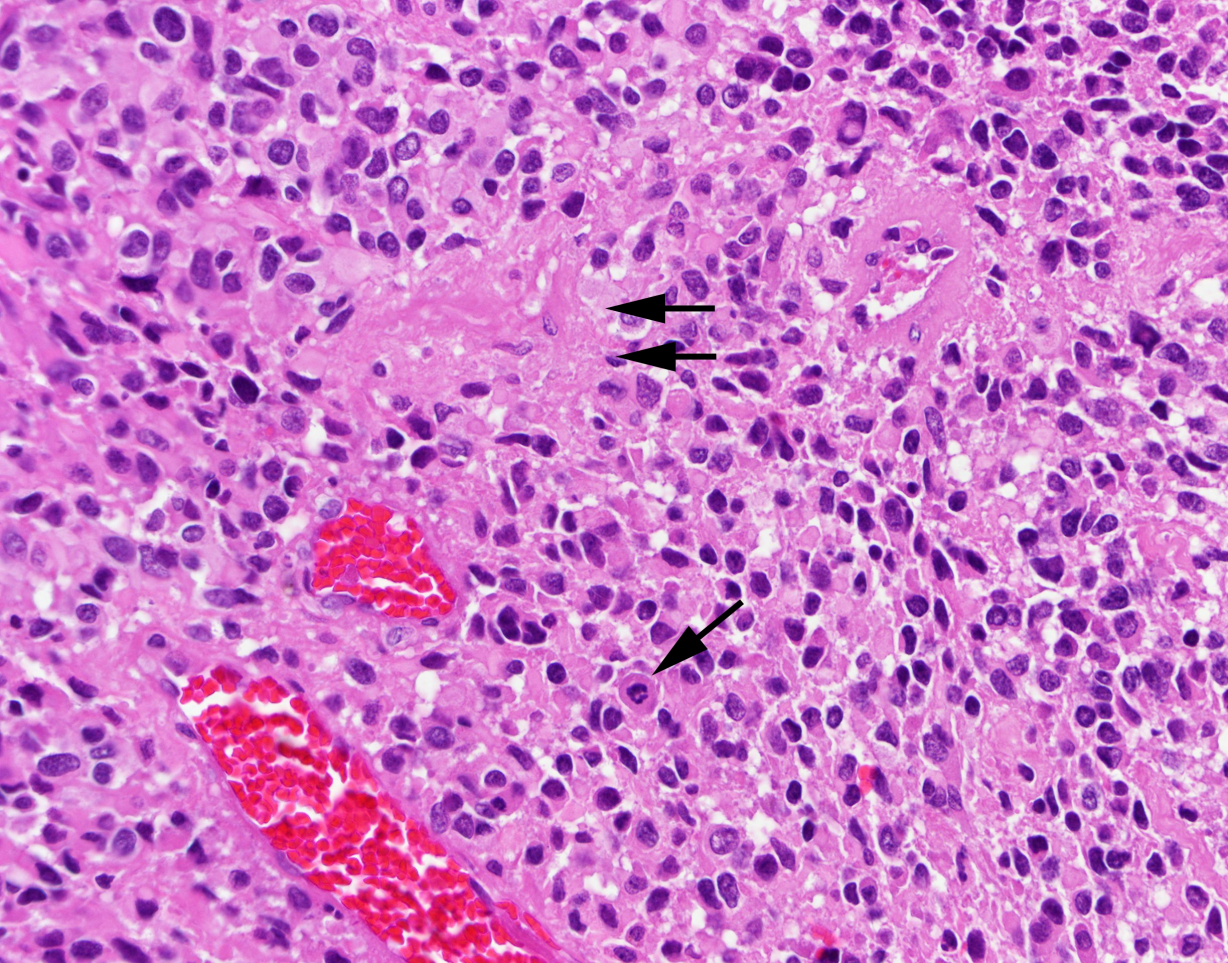
Hematoxylin-eosin (H&E), 400x Glioblastoma with mitosis (arrow) and focal necrosis (double arrows).

Final pathology revealed an isocitrate dehydrogenase (IDH)-1 wild-type glioblastoma (Figure 6).

## Discussion

We present a case of expressive aphasia in a strongly right-handed patient with a right temporal glioblastoma. The concept of crossed aphasia has been well-documented in patients with acute ischemic and hemorrhagic strokes [3]. For example, Alexander et al. [3] reported a case series of 10 patients in which five patients were right-handed and presented with crossed aphasia as a result of a right-sided cerebrovascular accident.

Crossed aphasia as a result of a brain neoplasm has rarely been discussed in the literature. To the best of our knowledge, there are only eight case reports published in English that describe such occurrences (Table 1) [2, 4-10].

**Table 1:** Results of literature review R: right; WHO: World Health Organization.

Author	Year	Number of Patients	Age (years)	Handedness	Presentation	Location of Tumor	Tumor
Mastronardi et al. [2]	1994	2	45; 69	Right-handed	Seizure and aphasia	R Temporal, R Temporoparietal,	WHO Grade IV glioma; WHO Grade III astrocytoma
Vassal et al. [4]	2010	3	27; 33; 45	Right-handed	Partial seizures, language disturbance	One R frontotemporoinsular, two R Temporal	Two WHO Grade II gliomas; one WHO Grade III glioma
Giovanogli [5]	1993	1	50	Right-handed	Wernicke aphasia, left spatial neglect	R Temporal	Glioblastoma
Riecker et al. [6]	2004	1	59	Right-handed	Partial seizure, mixed aphasia	R Temporal	WHO Grade III astrocytoma
Khateb et al. [7]	2004	1	54	Right-handed	Headache, aphasia, dysgraphia	R Frontal	Meningioma
Primavera et al. [8]	1993	1	63	Right-handed	Global aphasia, homonymous hemianopia, left hemiparesis	R Parieto-occipital	Glioblastoma
Martins et al. [9]	1987	1	15	Right-handed	Aphasia, alexia, agraphia, visuospatial disturbances	R Occipitotemporal	Oligodendroglioma
Larrabee [10]	1982	1	60	Right-handed	Aphasia	R Temporal	Likely metastatic

Many of these articles lack data on the handedness index and intraoperative details on the resection of the neoplasm. Probably one of the best illustrations of the occurrence of crossed aphasia as a result of brain tumors was illustrated by Vassal et al. [4], who discussed three patients with right-sided gliomas who were found to have language disorders on presentation or preoperative assessment. All of these patients were found to be 100% right-handed on the Edinburgh handedness inventory and underwent intraoperative awake cortical mapping for tumor resection. A subtotal resection was achieved in all cases; one patient experienced a transient dysphasia postoperatively which resolved after speech rehabilitation [4].

Matronardi et al. [2] reported two right-handed patients who presented with crossed aphasia and were found to have right-sided neoplasms. The first patient had a right-sided temporoparietal anaplastic astrocytoma, which was resected and later recurred, causing seizures, left-sided weakness, and expressive aphasia. The second patient had a subtotal resection of a right temporal low-grade glioma (which transformed into a Grade IV glioma over six years) and, following resection, developed a mixed aphasia [2].

Giovagnoli [5] reported a right-handed woman presenting with a crossed aphasia in the setting of a right-sided glioblastoma. The patient presented initially with acalculia, left hemianopsia, and Wernicke’s aphasia [5]. The patient was found to have a right posterior temporal lesion. Functional MRI was not available at that time to further characterize the lesion, but it appears to have some resemblance to the lesion seen in our report. Our patient, however, demonstrated expressive rather than receptive aphasia.

## Conclusions

Crossed aphasia as a manifestation of glioblastoma has rarely been reported in the literature. When such a scenario is encountered, further diagnostic workup, such as a functional MRI, neuropsychological testing, and diffusion tensor imaging with tractography, can assist in the further characterization of the lesion and its relation to critical speech regions. In such cases, we advocate for an awake resection with neurophysiological monitoring and cortical/subcortical stimulation to maximize the safety and efficacy of the resection.
